# Assessment of Endocyn on Dental Pulp Stem Cells (DPSCs): A Pilot Study of Endodontic Irrigant Effects

**DOI:** 10.3390/mps8010018

**Published:** 2025-02-11

**Authors:** Brennan Truman, Linda Ma, Samuel Stewart, Karl Kingsley, Victoria Sullivan

**Affiliations:** 1 Department of Advanced Education in Pediatric Dentistry, School of Dental Medicine, University of Nevada, 1700 West Charleston Blvd, Las Vegas, NV 89106, USAvictoria.sullivan@unlv.edu (V.S.); 2 Department of Clinical Sciences, School of Dental Medicine, University of Nevada, 1700 West Charleston Blvd, Las Vegas, NV 89106, USA; 3 Department of Biomedical Sciences, School of Dental Medicine, University of Nevada, 1001 Shadow Lane, Las Vegas, NV 89106, USA

**Keywords:** endodontic irrigant, Endocyn, dental pulp stem cells, disinfection method

## Abstract

Many endodontic procedures within the pediatric population are performed with patients aged 12 years and older, using intracanal irrigants to complement mechanical debridement for the removal of debris and to disinfect the root canal system. The use of antimicrobial irrigants that limit damage to the dental pulp are the goals of endodontic biomaterials research. Using an existing biorepository of dental pulp stem cells (DPSCs), Endocyn was evaluated in varying concentrations in proliferation and viability assays, and compared with positive (sodium hypochlorite or bleach) and negative (phosphate-buffered saline) controls. The DPSC viability was reduced in the range of −8.3% to −15.8%, *p* = 0.22 to *p* = 0.042, while the growth inhibition varied between −29.7% and −63%, *p* = 0.041 to *p* = 0.022. However, the RNA analysis revealed that no significant changes in biomarker mRNA expression (Nestin, NANOG, Sox2, Oct4, CD73, CD90, and CD105) were observed. These data demonstrated that all of the concentrations of Endocyn inhibited the DPSC viability and growth, although only high concentrations were statistically significant. Moreover, the administration of Endocyn did not alter the DPSC biomarker expression, which are novel and important findings not previously observed or reported that may assist with the development of clinical decision protocols and methods for the treatment of vital pulp tissue.

## 1. Introduction

Recent evidence has demonstrated that the incidence of minimally invasive dental procedures, such as pulpotomies and pulpectomies, has risen in recent years [1,2,3]. These increases may be due, in part, to the advances in both clinical methods and dental protocols to retain intact primary teeth whenever possible [4,5]. The analysis and evaluation of bioactive materials that allow for the retention and regeneration of vital dental pulp has been critical to the success of these endeavors among dental clinicians [6,7,8].

Much of the research has necessarily focused on the biomaterials used in dental pulp capping, which utilizes medicated biomaterials to place directly onto the dental pulp to promote healing and facilitate protection from microbial contamination [9,10,11]. However, other recent studies have demonstrated that alternative methods and clinical protocols may also be useful to increase the successful outcomes for these procedures among pediatric populations [12,13,14]. In fact, the effectiveness of vital pulp treatment may be equally dependent upon the disinfectants, irrigants, and associated antimicrobials used prior to the placement of the pulp capping biomaterials [15,16,17].

Traditional dental pulp irrigation methods have included the use of sodium hypochlorite, an effective oxidizing agent and antimicrobial agent more commonly referred to as bleach [18,19,20]. However, due to the inflammation and potential for damage to the dental pulp tissue, other alternatives, such as chlorhexidine gluconate or tetracycline antibiotics, have been utilized to increase the antimicrobial effects while limiting the potential for damage to the dental pulp [21,22,23]. Newer methods, including ozonated water and ultrasonication, have also been evaluated to determine not only the antimicrobial properties but also the potential effects on dental pulp tissues [24,25,26].

In addition, recent evidence has now demonstrated that hypochlorous acid, another form of chlorine-based disinfectant, may be more effective than sodium hypochlorite in clinical applications due to the ability to penetrate microbial cell walls while limiting the effects on the human host cells [27,28,29]. Endocyn is a recently developed, commercially available dental irrigant and disinfectant that has utilized a unique pH-neutral formulation of hypochlorous acid that remains stable over long periods of time—although research on the antimicrobial effectiveness has been limited [30]. Furthermore, only one study to date has evaluated the effects of Endocyn on dental pulp tissue, which was limited to the evaluation of stem cells from the apical papilla, with no evaluation of the potential effects on stem cells derived from the pulp chamber [31].

Many types of stem cells are present in various tissues of the oral cavity, including gingival mesenchymal stem cells, dental follicle stem cells, periodontal ligament stem cells, and stem cells the apical papilla [32,33,34]. However, pluripotent mesenchymal dental pulp stem cells (DPSCs) derived from the pulp chamber are the most likely to be affected by these types of irrigants, which have been the focus of recent dental biomaterials research, and studies of their potential effects on viability and growth, as well as biomarkers of pluripotency and differentiation potential [35,36]. Due to the lack of evidence regarding the effects on these specific DPSCs, the primary goal of this study was to evaluate the effects of Endocyn on DPSC phenotypes, including the viability, growth, and biomarker expression.

## 2. Methods

### 2.1. Study Approval for DPSC Lines

All of the study protocols were performed in accordance with the Declaration of Helsinki. This retrospective study of existing cell lines was reviewed and approved by the Institutional Review Board (IRB) and the Office for the Protection of Human Subjects (OPRS) at the University of Nevada, Las Vegas (UNLV). Protocol #171612-1 “Retrospective Analysis of Dental Pulp Stem Cells (DPSC) from the UNLV School of Dental Medicine (SDM) Pediatric and Clinical Population” was approved in February 2021, as previously described [35,36,37,38]. The protocol for the initial collection of DPSCs for this biorepository was originally reviewed and approved by the UNLV IRB and OPRS under Protocol OPRS#0907-3148 “Isolation of Non-Embryonic Stem Cells from Dental Pulp” in February 2010. In brief, voluntary UNLV-SDM clinic patients of record who provided informed consent or pediatric assent (if under 18 years of age) were included in the original study protocol. Any UNLV-SDM patients who declined to participate or declined to provide either informed consent or pediatric assent were excluded, as previously described [35,36,37,38].

The DPSC cell lines used in this study were obtained from the University of Nevada, Las Vegas (UNLV) School of Dental Medicine biorepository provided by Dr. Karl Kingsley, as previously described [35,36,37,38]. Each cell line was previously screened for the positive expression of mRNA for the clusters of differentiation (CD) CD73, CD90, and CD105, and the negative (lack of) expression of mRNA for CD45, as recommended by the International Society for Cellular Therapy (ISCT) [39,40]. Each DPSC cell line used in this study (*n* = 12) was thawed and centrifuged to remove the dimethyl sulfoxide (DMSO)-containing freezing media prior to the cell culture. The cells were then resuspended in Roswell Park Memorial Institute (RPMI) media, including the addition of bovine growth serum (BGS) and an antibiotic solution containing Penicillin-–Streptomycin (to concentrations of 10% and 1%, respectively), both obtained from Fisher Scientific (Fair Lawn, NJ, USA), and cultured in a Biosafety Level (BSL) 2 chamber at 37 °C supplemented with 5% carbon dioxide (CO_2_), as previously described [35,36,37,38]. The cell lines are included in Table 1.

### 2.2. Experimental Reagents

The negative control used in this study was phosphate-buffered saline or 1x PBS (#J61196-AP), with a pH of 7.4, and the positive control used was sterile sodium hypochlorite (5% bleach or 5000 ppm) in an aqueous solution, at a pH of 11.0 (#P005-03)—both were obtained from Fisher Scientific (Fair Lawn, NJ, USA). The experimental reagent Endocyn, at a pH of 7.1 (approximately 300 ppm hypochlorous acid), distributed by Sonoma pharmaceuticals (Woodstock, GA, USA), was purchased from New Line Medical (Breaux Bridge, LA, USA).

### 2.3. Growth Assays

Growth experiments were performed in 96-well flat-bottom Corning Costar assay plates from ThermoFisher Scientific (Waltham, MA, USA), as previously outlined [35,36]. In brief, suspensions (100 µL) of cells were plated at a standardized concentration of 1 × 10^5^ cells per mL and allowed to proliferate for three days, or 72 h (the primary endpoint), in the growth media with or without the experimental or control treatments. Negative (PBS) and positive (bleach) controls, as well as the experimental condition (Endocyn), were added at concentrations of 0% (baseline), 1:100 or 1%, 10:100 or 10%, and 50:100 or 50% at a 100 µL total volume, corresponding to other study protocols of endodontic irrigants assessing cellular phenotypes and antimicrobial activity [27,28,29,30,31]. Assay plates were fixed using 10% buffered formalin and subsequently stained using a Gentian Violet (1% *w*/*v*) solution obtained from Fisher Scientific (Fair Lawn, NJ, USA). The staining solution was aspirated and the experimental plates were then washed with 1x PBS to remove the non-cellular staining, and then were dried prior to processing using an ELx808 microplate reader from Eppendorf (Hamburg, Germany) at absorbance readings of A600 nm. Finally, the absorbance data were exported to Microsoft Excel (Redmond, WA, USA) for analysis. The percentage change from the baseline was calculated from the absorbance reading of the baseline (non-treated) cells compared with each experimental condition. Each assay was replicated in three independent experiments, with *n* = 8 wells per experimental condition (PBS, bleach, and Endocyn) and concentration (1:100 or 1%, 10:100 or 10%, and 50:500 or 50%).

### 2.4. Viability Assays

Cellular viability was assessed using the Trypan Blue dye exclusion method at three days, or 72 h (the primary endpoint), as previously described [37,38]. Briefly, an equal volume of Gibco 0.4% Trypan Blue, obtained from ThermoFisher Scientific (Waltham, MA, USA), was added to each experimental and control condition, and analyzed using a TC20 Cell Counter from BioRad Technologies (Hercules, CA, USA). The cell counts of the total and live cell numbers from each assay condition (positive control, negative control, and experimental reagent) were obtained and exported to Microsoft Excel (Redmond, WA, USA) for further analysis. Finally, the data for the viability assays were derived from three independent experiments, with *n* = 8 wells per experimental condition (PBS, bleach, and Endocyn) and concentration (1:100 or 1%, 10:100 or 10%, and 50:500 or 50%).

### 2.5. RNA Isolation and cDNA Synthesis

To evaluate any additional changes to the DPSC phenotypes, RNA was isolated from each DPSC under the control and Endocyn treatments, as outlined previously [35,36,37,38]. In brief, the cells were lysed using the TRIzol reagent from Invitrogen (Waltham, MA, USA) with the manufacturer-recommended protocol [35,36]. The cell lysate-containing solution (1.0 mL) was transferred to a sterile microcentrifuge tube with the addition of 0.2 mL of chloroform from Fisher Scientific (Fair Lawn, NJ, USA) and incubated for ten minutes. All of the samples were then centrifuged at a 10,000 relative centrifugal force (RCF) using a refrigerated 5424 Microcentrifuge from Eppendorf (Hamburg, Germany) for 15 min, as previously described [36,37]. The RNA-containing aqueous phase was transferred to a sterile microcentrifuge tube and an equal volume of isopropanol from Fisher Scientific (Fair Lawn, NJ, USA) was added to the precipitate the nucleic acids. All of the samples were then centrifuged once more to pellet the nucleic acids, and the pellet was subsequently washed with ethanol from Fisher Scientific (Fair Lawn, NJ, USA) before the final centrifugation. Each RNA pellet was resuspended using 0.1 mL of nuclease-free water (from Fisher Scientific). The quality and quantity of RNA was determined using a NanoDrop 2000 Spectrophotometer, also from Fisher Scientific (Fair Lawn, NJ, USA), using absorbances at A260 nm and A280 nm. The reverse transcription of the RNA was performed using the Verso 1-step RT-PCR kit (AB1454LDB) from ThermoFisher Scientific (Fair Lawn, NJ, USA) and the Mastercycler gradient thermal cycler from Eppendorf (Hamburg, Germany), using the protocol recommended by the manufacturer, as described previously [35,36,37,38]

### 2.6. Real-Time qPCR Screening

The screening of cDNA was performed using real-time quantitative polymerase reaction (qPCR) with the SYBR Green Master Mix kit from Fisher Scientific (Fair Lawn, NJ, USA), following the manufacturer-recommended protocols [25,26,27,28]. In brief, ABsolute SYBR Green Master Mix (12.5 µL), forward and reverse primers (1.5 µL each), nuclease-free water (7.5 µL), and cDNA from each DPSC isolate (1.0 µL) were combined and processed using the QuantStudio real-time PCR system from Applied Biosystems (Waltham, MA, USA). The reaction settings included enzyme activation for 15 min at 95 °C, followed by 40 cycles of denaturation for 15 s at 95 °C, then annealing at a primer-pair-specific temperature for 30 s, and a final extension at 72 °C for 30 s using the following validated primer sets, as described previously [35,36,37,38].
Positive control primer:Glyceraldehyde 3-phosphate dehydrogenase (GAPDH)GAPDH forward primer5′-ATC TTC CAG GAG CGA GAT CC-3′GAPDH reverse primer5′-ACC ACT GAC ACG TTG GCA GT-3′ISCT (MSC) validation primers:
CD45 forward primer5′-CAT ATT TAT TTT GTC CTT CTC CCA-3′CD45 reverse primer5′-GAA AGT TTC CAC GAA CGG-3′CD73 forward primer5′-AGT CCA CTG GAG AGT TCC TGC A-3′CD73 reverse primer5′-TGA GAG GGT CAT AAC TGG GCA C-3′CD90 forward primer5′-ATG AAC CTG GCC ATC AGC A-3′CD90 reverse primer5′-GTG TGC TCA GGC ACC CC-3′CD105 forward primer5′-CCA CTA GCC AGG TCT CGA AG-3′CD105 reverse primer5′-GAT GCA GGA AGA CAC TGC TG-3′Stem cell biomarker primers:
Nestin forward primer5′-CGT TGG AAC AGA GGT TGG AG-3′Nestin reverse primer5′-TCC TGA AAG CTG AGG GAA G-3′NANOG forward primer5′-GCT GAG ATG CCT CAC ACG GAG-3′NANOG reverse primer5′-TCT GTT TCT TGA CTG GGA CCT TGT C-3′Oct-4 forward primer5′-TGG AGA AGG AGA AGC TGG AGC AAA A-3′Oct-4 reverse primer5′-GGC AGA TGG TCG TTT GGC TGA ATA-3′Sox-2 forward primer5′-ATG GGC TCT GTG GTC AAG TC-3′Sox-2 reverse primer5′-CCC TCC CAA TTC CCT TGT AT-3′Alkaline phosphatase (ALP)
ALP forward primer5′-CAC TGC GGA CCA TTC CCA CGT CTT-3′ALP reverse primer5′-GCG CCT GGT AGT TGT TGT GAG CAT-3′Dentin sialophosphoprotein (DSPP)
DSPP forward primer5′-CAA CCA TAG AGA AAG CAA ACG CG-3′DSPP reverse primer5′-TTT CTG TTG CCA CTG CTG GGA C-3′

Screening data from the qPCR targets were normalized to the internal positive (endogenous) control, glyceraldehyde 3-phosphate dehydrogenase (GAPDH), which was facilitated using the QuantStudio, Version 5 software from Applied Biosystems (Waltham, MA, USA), in order to facilitate corrections for sample-to-sample variations with respect to the RT-PCR efficiency, as previously described [37,38].

### 2.7. Statistical Analysis

Experimental data from the growth and viability assays were analyzed using two-tailed Student’s *t*-tests from Microsoft Excel (Redmond, WA, USA), which are appropriate for continuous parametric analysis. Differences between the baseline measurements (no reagent) and the controls (positive and negative) were compared with the experimental reagent (Endocyn). Statistically significant differences that were identified by this analysis were also verified using an analysis of variance (ANOVA) and the Prism 9 version software from GraphPad (Boston, MA, USA) due to the possibility of Type I errors with the analysis of multiple two-way t-tests. Statistical significance was determined with an alpha level set at *p* = 0.05, as previously described [35,36,37,38].

## 3. Results

**Figure 1:**
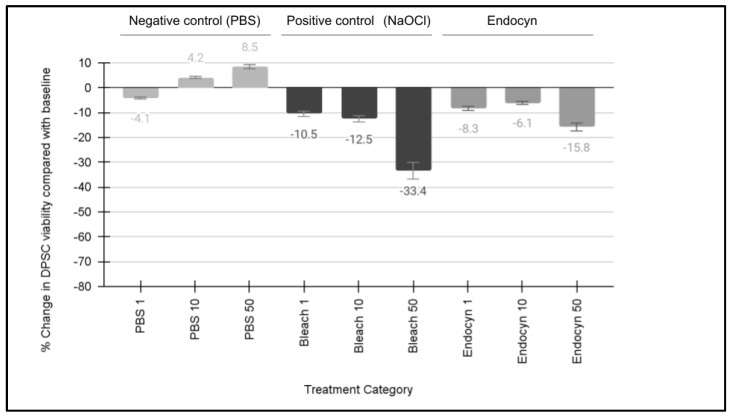
Effects of Endocyn on the DPSC viability. Minor changes to the DPSC viability were induced by PBS (negative control) at 1% (−4.1%), 10% (4.2%), and 50% (8.5%), *p* > 0.05. However, significant differences were observed with the NaOCl (positive control) at 1% (−10.5%), 10% (−12.5%), and 50% (−33.4%), *p* < 0.05. Endocyn induced minor changes to the viability at 1% (−8.3%) and 10% (−6.1%), *p* > 0.05, but significant reductions at the highest concentration of 50% (−15.8%), *p* = 0.042.

An analysis of the effects of Endocyn compared with the positive and negative controls on the DPSC viability was performed (Figure 1). These data demonstrated that no significant differences in cellular viability were observed with the negative control (PBS) at any of the concentrations evaluated, including 1:100 or 1% (−4.1%, *p* = 0.788), 10:100 or 10% (4.2%, *p* = 0.711), or 50:100 or 50% (8.5%, *p* = 0.231). However, significant differences were observed with the positive control (NaOCl or bleach) at each of the same concentrations of 1:100 or 1% (−10.5% *p* = 0.050), 10:100 or 10% (−12.5%, *p* = 0.049), and 50:100 or 50% (−33.4%, *p* = 0.033). An analysis of the results from the Endocyn trials revealed differential results, with no significant differences in viability at the lowest concentrations of 1:100 or 1% (−8.3%, *p* = 0.22) or 10:100 or 10% (−6.1%, *p* = 0.513), but a significant reduction in viability was observed at the highest concentration of 50:100 or 50% (−15.8%, *p* = 0.042).

**Figure 2:**
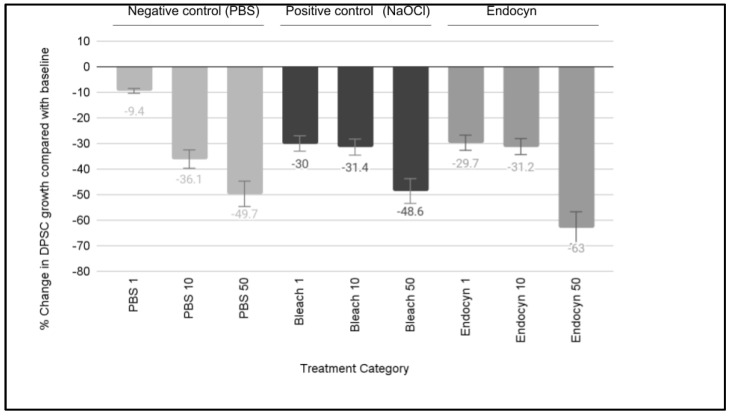
Effects of Endocyn on the DPSC growth. The DPSC growth was inhibited by PBS (negative control) at 1% (−9.4%), 10% (−36.1%), and 50% (−49.7%), *p* < 0.05, as well as NaOCl (positive control) over the range of 1% (−30.0), 10% (−31.4%), and 50% (−48.6%), *p* < 0.05. Endocyn also inhibited the DPSC growth at all concentrations, including 1% (−29.7%), 10% (−31.2%), and 50% (−63.0%), *p* < 0.05.

A comparison of the effects of Endocyn with the positive and negative controls on DPSC growth was also performed (Figure 2). These results revealed that significant differences in cellular growth were observed with the negative control (PBS) at all of the concentrations evaluated, including 1:100 or 1% (−9.4%, *p* = 0.050), 10:100 or 10% (−36.1%, *p* = 0.031), or 50:100 or 50% (−49.7%, *p* = 0.012). Significant reductions in growth were also observed with the negative control (NaOCl or bleach) at each of the same concentrations of 1:100 or 1% (−30.0% *p* = 0.038), 10:100 or 10% (−31.4%, *p* = 0.036), and 50:100 or 50% (−48.6%, *p* = 0.015). The administration of Endocyn induced similar results, with significant reductions in growth observed at all concentrations, including 1:100 or 1% (−29.7%, *p* = 0.041), 10:100 or 10% (−31.2%, *p* = 0.037), and 50:100 or 50% (−63.0%, *p* = 0.012).

**Figure 3:**
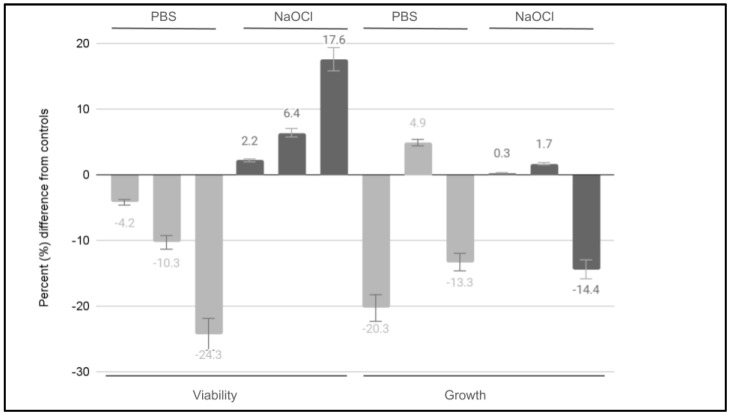
Comparison of Endocyn-induced changes with the positive and negative controls. Endocyn reduced the cellular viability compared with PBS at 1% (−4.2%, *p* = 0.771), 10% (−10.3, *p* = 0.050), and 50% (−24.3%, *p* = 0.042), but increased the cellular viability compared with sodium hypochlorite at these concentrations of 1% (2.2%, *p* = 0.81), 10% (6.4%, *p* = 0.52), and 50% (17.6%, *p* = 0.0441). Significant reductions in growth were also observed at the low and high concentrations of 1% (−20.3%, *p* = 0.0378) and 50% (−13.3%, *p* = 0.0412), but increased moderately at the mid-level concentration of 10% (4.9%, *p* = 0.681). In addition, slight increases in the growth compared with sodium hypochlorite were observed at both 1% (0.3%, *p* = 0.911) and 10% (1.7%, *p* = 0.889), but were significantly reduced at the highest concentration of 50% (−14.4%, *p* = 0.0437).

To more accurately evaluate the potential effects on DPSCs, the differences between Endocyn-induced changes from the baseline were compared with the positive and negative controls (Figure 3). These data clearly demonstrated the differential effects of Endocyn on the viability compared with the experimental controls. For example, although Endocyn reduced the cellular viability compared with PBS at 1:100 or 1% (−4.2%, *p* = 0.771), 10:100 or 10% (−10.3, *p* = 0.050), and 50:100 or 50% (−24.3%, *p* = 0.042), it increased the cellular viability compared with sodium hypochlorite at these concentrations of 1:100 or 1% (2.2%, *p* = 0.81), 10:100 or 10% (6.4%, *p* = 0.52), and 50:100 or 50% (17.6%, *p* = 0.0441). The differential results were also observed among the DPSCs with the comparisons of Endocyn and the experimental controls. More specifically, significant reductions in growth compared with PBS were observed at the low and high concentrations of 1:100 or 1% (−20.3%, *p* = 0.0378) and 50:100 or 50% (−13.3%, *p* = 0.0412), but increased moderately at the mid-level concentration of 10:100 or 10% (4.9%, *p* = 0.681). In addition, slight increases in the growth compared with sodium hypochlorite were observed with Endocyn at both the 1:100 or 1% (0.3%, *p* = 0.911) and 10:100 or 10% (1.7%, *p* = 0.889) concentrations, but were significantly reduced at the highest concentration of 50:100 or 50% (−14.4%, *p* = 0.0437). More detailed information for each treatment and cell line can be found in Appendix A.

**Figure 4:**
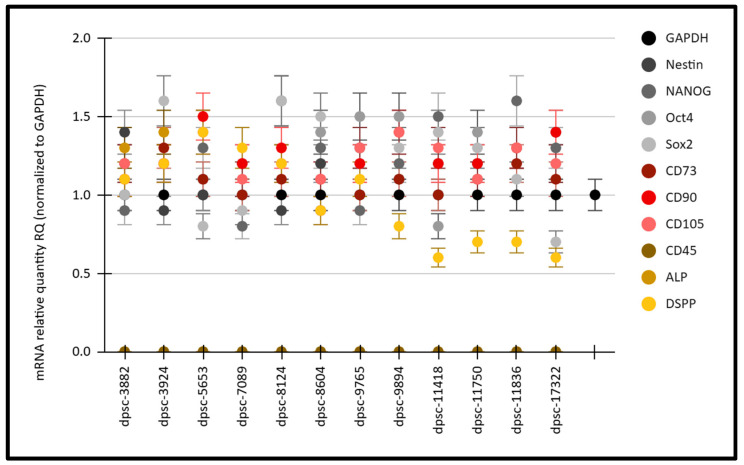
Analysis of the mRNA biomarker expression for the DPSC cell lines. The mRNA expression normalized to GAPDH was consistent for the MSC biomarkers Nestin (RQ: 1.18), NANOG (RQ: 1.23), Oct4 (RQ: 1.27), and Sox2 (RQ: 1.2), as well as the ISCT biomarkers CD73 (RQ: 1.09), CD90 (RQ: 1.25), and CD105 (RQ: 1.23). More variability was observed with the osteogenic biomarkers DSPP (RQ: 0.97) and ALP (RQ: 1.35).

An analysis of the mRNA expression for key DPSC and MSC markers was performed on all DPSC cell lines (Figure 4). These data revealed that the mRNA relative quantity (RQ) normalized to the positive control glyceraldehyde 3-phosphate dehydrogenase (GAPDH) mRNA expression was fairly consistent among all of the DPSC lines for the MSC biomarkers Nestin (average: 1.18; range: 0.9 to 1.4), NANOG (average: 1.23; range: 0.8 to 1.5), Oct4 (average: 1.27; range: 0.8 to 1.4), and Sox2 (average: 1.2; range: 0.7 to 1.4). In addition, the ISCT biomarkers also demonstrated consistent expression among all of the DPSC lines, including CD73 (average: 1.09; range: 0.9 to 1.2), CD90 (average: 1.25; range: 1.1 to 1.5), and CD105 (average: 1.23; range: 1.1 to 1.4). More variability was observed with the osteogenic and osteoblast-associated targets dentin sialophosphoprotein (DSPP; average: 0.97; range: 0.6 to 1.4) and alkaline phosphatase (ALP; average: 1.35; range: 1.1 to 1.5).

**Figure 5:**
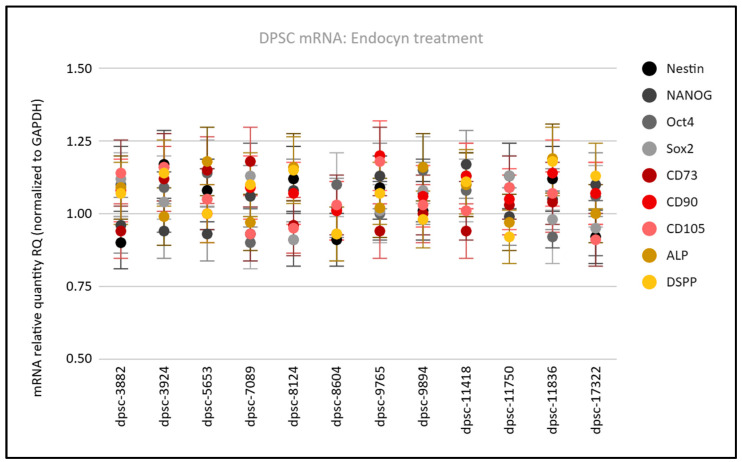
Analysis of the Endocyn effects on mRNA biomarker expression for the DPSC cell lines. Following the Endocyn treatment, the mRNA expression normalized to GAPDH and, compared with the baseline (untreated cells), was consistent for the MSC biomarkers Nestin (RQ: 1.04), NANOG (RQ: 1.04), Oct4 (RQ: 1.05), and Sox2 (RQ: 1.03), the ISCT biomarkers CD73 (RQ: 1.04), CD90 (RQ: 1.08), and CD105 (RQ: 1.04), and the osteogenic biomarkers DSPP (RQ: 1.05) and ALP (RQ: 1.07).

To determine if the Endocyn treatment altered the mRNA expression among the DPSC cell lines, RNA was collected following each experiment and screened using qPCR (Figure 5). This analysis revealed that the mRNA relative quantity (RQ) normalized to the internal positive control GAPDH was not significantly altered compared with the baseline (untreated cells) among the DPSC lines, more specifically, the MSC biomarkers Nestin (average: 1.04; range: 0.92 to 1.13), NANOG (average: 1.04; range: 0.93 to 1.17), Oct4 (average: 1.05; range: 0.91 to 1.15), and Sox2 (average: 1.03; range: 0.95 to 1.12). The ISCT biomarkers were also not significantly altered by the Endocyn treatment, including CD73 (average: 1.03; range: 0.93 to 1.18), CD90 (average: 1.08; range: 1.01 to 1.2), and CD105 (average: 1.04; range: 0.93 to 1.17). Similar results were observed with the osteogenic and osteoblast-associated targets DSPP (average: 1.06; range: 0.92 to 1.16) and ALP (average: 1.07; range: 1.01 to 1.15).

## 4. Discussion

Although many types of pluripotent mesenchymal stem cells are present in the oral cavity, DPSCs derived from the pulp chamber are the most likely to be affected by endodontic irrigants, and were thus the primary focus of this study [41,42]. As only one previous study had evaluated the stem cells of the apical papilla (SCAP), these data demonstrated the effects of Endocyn using multiple DPSCs derived from the pulp chamber [35,36,37,38]. Therefore, these data may be the first to evaluate the effects of this endodontic irrigant on DPSC phenotypes, including viability and growth.

These data revealed that, although the Endocyn administration was sufficient to reduce DPSC growth, similar to the positive control (sodium hypochlorite), the effects on the DPSC viability were much less robust [43,44]. This may represent a significant finding, as the goal of endodontic treatments is not only to provide debris removal and site-specific antimicrobial medication, but also to retain as much vital pulp tissue as possible so as to facilitate healing and wound repair [45,46]. As sodium hypochlorite may be considered the clinical standard for antimicrobial endodontic irrigants, any alternative that provides similar antimicrobial effects with lower levels of toxicity and damage to human cells could be an important component of clinical treatment protocols [47,48].

In addition, this study may be among the first to demonstrate that the effects of Endocyn on host cells are similar to those observed with other formulations of hypochlorous acid [49,50]. Recent studies have called for the development and incorporation of biomaterials utilizing hypochlorous acid for these types of dental procedures because of the increased antimicrobial potential, as well as the milder and less toxic effects on host cells and tissues [51,52]. The results from this study support this research and other findings that suggest DPSC regeneration potential may be positively associated with the use of these bioactive materials in endodontic protocols [53,54].

Furthermore, the detailed analysis of Endocyn on both DPSC cell proliferation and viability demonstrated that the effects were similar across the entire range of stem cells, which were originally derived from different patients of different ages [35,36,37,38]. This may suggest that the magnitude and direction of DPSC responses to Endocyn may be well represented by the data from this type of initial pilot study—although, more studies will be needed to confirm these observations [55,56]. Finally, these analyses may be the first to determine that Endocyn administration does not significantly affect MSC biomarkers (CD73, CD90, and CD105) or DPSC pluripotency-related mRNA expression (Nestin, NANOG, Sox2, and Oct4) [35,36,37,38]. These data are important to aid clinicians and pediatric dentists, as they strive to conserve more primary teeth through the use of these minimally invasive procedures, and as the development and validation of clinical protocols and methods that improve patient outcomes becomes ever more critical [57,58].

Despite the significance of these findings, there are limitations that should be considered when evaluating the preliminary evidence from this type of in vitro pilot study. First, and most important, this study utilized DPSCs from an existing biorepository for laboratory-based evidence and does not represent the type of clinical research that would determine the effects of Endocyn in real-world patient settings [59,60]. In addition, due to the nature of this study, these data were also limited in the time frame evaluated, and longer-term effects on cell viability and growth were not possible to evaluate [61,62]. Furthermore, no data were available to determine the age of the DPSC donor, information which may be important for future studies to determine if DPSCs from hosts of different ages respond differentially to Endocyn in an age-dependent manner [63,64]. Finally, this study did not assess the antimicrobial properties of Endocyn specifically—although, other recent studies of hypochlorous acid have suggested it may be an effective clinical application due to the increased ability for this compound to penetrate microbial cell walls, while simultaneously reducing toxicity and inflammation on human host cells through the unique pH-neutral formulation of this product [27,28,29]. In fact, only one current study has evaluated the antimicrobial properties of Endocyn, which demonstrated that the effectiveness against both Gram-positive and Gram-negative bacteria was similar to sodium hypochlorite [30].

## 5. Conclusions

This pilot study provides some of the first comprehensive data and analysis of DPSC responses to Endocyn, a commercial disinfectant and irrigation product used in endodontic procedures that include pulpotomy, pulpectomy, and root canals. These data revealed similar effects on DPSC growth as the positive control (sodium hypochlorite), but with less toxic effects on cellular viability—an important consideration for vital pulp treatment and therapy. These data may also provide some of the first information regarding the maintenance of MSC and DPSC biomarker expression, a potentially important clinical consideration of dental therapy that may have the potential to improve DPSC viability and survival among patients undergoing vital pulp treatments and therapies.

## Figures and Tables

**Table 1:** DPSC cell lines.

Cell Line	Doubling Time (Growth Rate)	Viability (Following Thaw)
dpsc-3882	1.8 days	88%
dpsc-3924	2.2 days	86%
dpsc-5653	1.9 days	87%
dpsc-7089	1.7 days	82%
dpsc-8124	4.4 days	73%
dpsc-8604	4.1 days	71%
dpsc-9765	2.1 days	79%
dpsc-9894	5.2 days	76%
dpsc-11418	8.4 days	63%
dpsc-11750	9.9 days	62%
dpsc-11836	11.2 days	67%
dpsc-17322	10.6 days	66%

## Data Availability

The data presented in this study are presented in full and any other supporting data are available upon request from the corresponding author.

## Abbreviations

The following abbreviations are used in this manuscript:

NaOCl	Sodium hypochlorite
DPSC	Dental pulp stem cell
UNLV	University of Nevada, Las Vegas
DMSO	Dimethyl sulfoxide
PBS	Phosphate-buffered saline
CD	Cluster of differentiation
ISCT	International Society for Cellular Therapy
BGS	Bovine growth serum

## Appendix A

More comprehensive details corresponding to each specific DPSC cell line in response to the predictor (independent) variables were compiled and are summarized to provide the range of DPSC responses observed in this study (Table A1).

**Table A1:** DPSC responses to experimental variables.

	Experimental Condition Observed Response	Statistical Analysis
dpsc-3882 dpsc-3924 dpsc-5653 dpsc-7089 dpsc-8124 dpsc-8604 dpsc-9765 dpsc-9894 dpsc-11418 dpsc-11750 dpsc-11836 dpsc-17322	Viability [1% PBS] −3.7% −3.4% −4.6% −4.3% −4.1% −4.0% −3.2% −4.4% −3.9% −4.2% −4.6% −4.5%	Average: −4.1% Standard deviation: 0.45 Range: −3.2% to −4.6%
dpsc-3882 dpsc-3924 dpsc-5653 dpsc-7089 dpsc-8124 dpsc-8604 dpsc-9765 dpsc-9894 dpsc-11418 dpsc-11750 dpsc-11836 dpsc-17322	Growth [1% PBS] −10.8% −11.4% −8.5% −7.7% −11.4% −9.9% −9.1% −7.5% −8.6% −9.3% −9.1% −9.4%	Average: −9.4% Standard deviation: 1.29 Range: −7.5% to −11.4%
dpsc-3882 dpsc-3924 dpsc-5653 dpsc-7089 dpsc-8124 dpsc-8604 dpsc-9765 dpsc-9894 dpsc-11418 dpsc-11750 dpsc-11836 dpsc-17322	Viability [10% PBS] 3.4% 4.5% 3.9% 4.1% 3.9% 4.5% 4.4% 4.1% 4.6% 4.2% 3.9% 4.4%	Average: 4.2% Standard deviation: 0.35 Range: 3.4% to 4.5%
dpsc-3882 dpsc-3924 dpsc-5653 dpsc-7089 dpsc-8124 dpsc-8604 dpsc-9765 dpsc-9894 dpsc-11418 dpsc-11750 dpsc-11836 dpsc-17322	Growth [10% PBS] −40.2% −32.8% −35.4% −37.2% −41.2% −40.0% −38.4% −32.5% −37.4% −35.6% −31.2% −31.3% −36.2%	Average: −36.1% Standard deviation: 3.54 Range: −31.2% to −41.2%
dpsc-3882 dpsc-3924 dpsc-5653 dpsc-7089 dpsc-8124 dpsc-8604 dpsc-9765 dpsc-9894 dpsc-11418 dpsc-11750 dpsc-11836 dpsc-17322	Viability [50% PBS] 7.7% 10.6% 9.3% 8.7% 8.2% 9.1% 8.9% 7.8% 7.1% 6.8% 8.6% 8.8%	Average: 8.5% Standard deviation: 1.03 Range: 7.7% to 10.6%
dpsc-3882 dpsc-3924 dpsc-5653 dpsc-7089 dpsc-8124 dpsc-8604 dpsc-9765 dpsc-9894 dpsc-11418 dpsc-11750 dpsc-11836 dpsc-17322	Growth [50% PBS] −45.5% −53.1% −48.3% −51.2% −53.3% −46.4% −43.2% −49.8% −53.2% −55.1% −48.2% −49.0% −49.7%	Average: −49.7% Standard deviation: 3.61 Range: −45.5% to −55.1%
dpsc-3882 dpsc-3924 dpsc-5653 dpsc-7089 dpsc-8124 dpsc-8604 dpsc-9765 dpsc-9894 dpsc-11418 dpsc-11750 dpsc-11836 dpsc-17322	Viability [1% NaOCl] −11.4% −12.3% −14.6% −8.8% −9.4% −9.3% −9.4% −10.6% −11.4% −11.7% −8.9% −8.3%	Average: −10.5% Standard deviation: 1.84 Range: −8.3% to −14.6%
dpsc-3882 dpsc-3924 dpsc-5653 dpsc-7089 dpsc-8124 dpsc-8604 dpsc-9765 dpsc-9894 dpsc-11418 dpsc-11750 dpsc-11836 dpsc-17322	Growth [1% NaOCl] −25.5% −28.5% −31.2% −36.7% −33.4% −29.9% −32.4% −27.8% −28.3% −31.6% −28.9% −25.2% −30.0%	Average: −30.0% Standard deviation: 3.31 Range: −25.2% to −33.4%
dpsc-3882 dpsc-3924 dpsc-5653 dpsc-7089 dpsc-8124 dpsc-8604 dpsc-9765 dpsc-9894 dpsc-11418 dpsc-11750 dpsc-11836 dpsc-17322	Viability [10% NaOCl] −13.4% −10.8% −15.6% −11.4% −10.2% −13.3% −11.4% −13.6% −15.1% −11.8% −12.1% −11.8%	Average: −12.5% Standard deviation: 1.67 Range: −10.2% to −15.6%
dpsc-3882 dpsc-3924 dpsc-5653 dpsc-7089 dpsc-8124 dpsc-8604 dpsc-9765 dpsc-9894 dpsc-11418 dpsc-11750 dpsc-11836 dpsc-17322	Growth [10% NaOCl] −35.9% −31.7% −29.8% −28.6% −31.7% −33.5% −35.5% −32.4% −32.5% −29.5% −27.6% −27.9%	Average: −31.4% Standard deviation: 2.77 Range: −27.6% to −35.9%
dpsc-3882 dpsc-3924 dpsc-5653 dpsc-7089 dpsc-8124 dpsc-8604 dpsc-9765 dpsc-9894 dpsc-11418 dpsc-11750 dpsc-11836 dpsc-17322	Viability [50% NaOCl] −39.9% −33.1% −32.6% −37.5% −29.6% −32.7% −33.1% −31.2% −32.4% −29.3% −35.6% −33.5%	Average: −33.4% Standard deviation: 3.05 Range: −29.3% to −39.9%
dpsc-3882 dpsc-3924 dpsc-5653 dpsc-7089 dpsc-8124 dpsc-8604 dpsc-9765 dpsc-9894 dpsc-11418 dpsc-11750 dpsc-11836 dpsc-17322	Growth [50% NaOCl] −55.2% −48.2% −49.7% −48.2% −51.1% −55.3% −44.8% −43.7% −41.7% −47.4% −49.2% −48.1%	Average: −48.6% Standard deviation: 4.09 Range: −41.7% to −55.3%
dpsc-3882 dpsc-3924 dpsc-5653 dpsc-7089 dpsc-8124 dpsc-8604 dpsc-9765 dpsc-9894 dpsc-11418 dpsc-11750 dpsc-11836 dpsc-17322	Viability [1% Endocyn] −10.2% −9.9% −7.4% −7.7% −9.4% −6.9% −9.2% −9.4% −7.8% −7.6% −7.1% −6.8%	Average: −8.3% Standard deviation: 1.24 Range: −6.9% to −10.2%
dpsc-3882 dpsc-3924 dpsc-5653 dpsc-7089 dpsc-8124 dpsc-8604 dpsc-9765 dpsc-9894 dpsc-11418 dpsc-11750 dpsc-11836 dpsc-17322	Growth [1% Endocyn] −33.4% −27.2% −28.6% −33.8% −35.9% −34.5% −26.5% −27.8% −29.3% −31.1% −25.5% −22.4%	Average: −29.7% Standard deviation: 4.11 Range: −22.4% to −35.9%
dpsc-3882 dpsc-3924 dpsc-5653 dpsc-7089 dpsc-8124 dpsc-8604 dpsc-9765 dpsc-9894 dpsc-11418 dpsc-11750 dpsc-11836 dpsc-17322	Viability [10% Endocyn] −5.5% −7.3% −5.5% −5.9% −6.3% −6.6% −5.2% −5.7% −6.2% −7.2% −5.9% −6.2%	Average: −6.1% Standard deviation: 0.66 Range: −5.2% to −7.3%
dpsc-3882 dpsc-3924 dpsc-5653 dpsc-7089 dpsc-8124 dpsc-8604 dpsc-9765 dpsc-9894 dpsc-11418 dpsc-11750 dpsc-11836 dpsc-17322	Growth [10% Endocyn] −32.5% −34.7% −28.7% −31.2% −27.6% −32.4% −33.1% −36.4% −32.4% −28.5% −27.4% −29.5%	Average: −31.2% Standard deviation: 2.88 Range: −27.4% to−36.4%
dpsc-3882 dpsc-3924 dpsc-5653 dpsc-7089 dpsc-8124 dpsc-8604 dpsc-9765 dpsc-9894 dpsc-11418 dpsc-11750 dpsc-11836 dpsc-17322	Viability [50% Endocyn] −11.3% −18.2% −14.3% −15.5% −18.6% −18.4% −17.5% −16.4% −16.9% −11.9% −12.3% −11.2%	Average: −15.8% Standard deviation: 2.89 Range: −11.2% to −18.6%
dpsc-3882 dpsc-3924 dpsc-5653 dpsc-7089 dpsc-8124 dpsc-8604 dpsc-9765 dpsc-9894 dpsc-11418 dpsc-11750 dpsc-11836 dpsc-17322	Growth [50% Endocyn] −55.8% −64.6% −67.5% −68.1% −61.2% −63.2% −55.5% −65.4% −66.6% −61.2% −67.3% −59.0%	Average: −63.0% Standard deviation: 4.44 Range: −55.5% to −68.1%
